# Clinical and ultrasonographic features associated to response to intraarticular corticosteroid injection. A one year follow up prospective cohort study in knee osteoarthritis patient with joint effusion

**DOI:** 10.1371/journal.pone.0191342

**Published:** 2018-01-19

**Authors:** Joan Calvet, Cristóbal Orellana, Carlos Galisteo, María García-Manrique, Noemí Navarro, Assumpta Caixàs, Marta Larrosa, Jordi Gratacós

**Affiliations:** 1 Rheumatology Department, Parc Taulí Sabadell University Hospital. I3PT Research Institute (UAB), Sabadell, Barcelona, Spain; 2 Endocrinology and Nutrition Department, Parc Taulí Sabadell University Hospital. I3PT Research Institute (UAB), Sabadell, Barcelona, Spain; Augusta University, UNITED STATES

## Abstract

**Objectives:**

Intraarticular injection is used for pain relief in knee osteoarthritis (OA), but there is not a well defined profile of patient who could get more benefit from it. The purpose of this study was to evaluate the frequency of pain relief at one year after corticosteroids intraarticular injection and to identify clinical factors associated to response in patients with knee osteoarthritis with joint effusion.

**Methods:**

One-year prospective cohort study of patients with knee OA with joint effusion confirmed by ultrasound. An intraarticular injection was performed following a clinical protocol. Anthropometric measurements, laboratory parameters, clinical severity, ultrasound parameters and radiological severity were collected. Response regarding pain and presence of synovial fluid on ultrasound at one month and at one year were evaluated. Clinical responder were consider in subjects with enough improvement to carry out normal daily activities with pain VAS<40mm.

**Results:**

One hundred and thirty-two patients were included.A significant number of patients (61.4%) improved pain at one year following the protocol established in this study. Pain and ultrasound synovial fluid at one month appeared to predict the response at one year. The Lequesne index and the percentage of body fat were independently associated to pain at one year while the Lequesne index and ultrasound synovial hypertrophy were independently related to the presence of synovial fluid at one year.

**Conclusions:**

The status regarding pain or ultrasound synovial fluid at one month after an intraarticular joint injection appeared to predict the status at one year in patients with knee osteoarthritis and synovial effusion.

## Introduction

Osteoarthritis (OA) is a disabling musculoskeletal disorder and knee OA the most prevalent condition[1]. OA prevalence increases with age and sometimes patients suffering from symptomatic knee OA have cardiovascular comorbidities limiting treatment options[2–4]. Treatments include acetaminophen, NSAIDs (nonsteroidal anti-inflammatory drugs), low-power opioids and SYSADOAs (symptomatic slow-action drugs for osteoarthritis), but it is not unusual that patients do not achieve an adequate improvement. In daily clinical practice intraarticular corticosteroid injections (IACI) are often used as a treatment for pain relief[5] but, although 95% of rheumatologists use IACI as a therapeutic technique[6], there is not a consensus on the pattern of administration. Different societies recommend IACI of long-acting corticosteroid as a treatment option for knee osteoarthritis [7, 8] and especially for knee pain flares if accompanied by effusion[9]. Different review shave pointed to a clinically significant short effect in pain, but the evidence did not support the use of IACI for long-term pain relief[10–12]. There are few randomized clinical trials focused on the long-term efficacy of IACI and most of them with a limited time of follow-up[13, 14]. Some studies were carried out to investigate predictors of IACI response but the conclusions were not robust enough to make recommendations[15]. Some previous data conclude that patients with a more inflammatory profile could show a better response to IACI[16, 17]. Taking into account the role of inflammation in knee OA[18, 19], patients with persistent knee effusion could define a special phenotype[20]. Our purpose was to evaluate the frequency of patients with pain relief at one year of follow-up in a homogeneous group of knee OA patients with joint effusion in daily clinical practice using a preestablished protocol of IACI for flares, and to identify clinical factors associated to a better intraarticular injection response at one year of follow-up.

## Methods and materials

### Patients and design

Prospective cohort study of one-year follow-up, with systematic inclusion of patients with symptomatic primary knee OA according to ACR criteria[21], visited in a monographic OA consultation at our hospital, aged 50–85 years old and showing persistent joint effusion on physical examination confirmed by ultrasound (≥4mm on midline suprapatellar line). Symptomatic OA was defined as pain intensity ≥4cm on a 10-cm visual analogical scale despite the use of prescribed analgesic drugs for at least three months. Exclusion criteria were secondary OA and intraarticular corticoids or hyaluronic acid injections in the last three or six months, respectively. Recruitment period comprised October 2013 to October 2015. The investigation was conducted according to the principles expressed in the Declaration of Helsinki. Written informed consent was obtained from all participants. The study was approved by Local Ethics Committee, Consorci Corporació Sanitària Parc Taulí (2013/591).

### Assessments

Variables collected were: age and knee OA symptom duration. Medical history with anthropometric measurements: weight (kg), height (cm), body mass index (BMI, kg/cm^2^),waist circumference (WC, cm) and percentage of body fat measured by bioelectric impedanciometry (TANITA BC-418MA bio logica) following standard protocol. Obesity was considered if BMI ≥30. The Lequesne algofunctional index, a validated questionnaire to assess clinical severity was used at the time of baseline IACI[22]. Radiographic severity was evaluated by antero-posterior knee X-ray examination in standing position and graded according to the Kellgren-Lawrence scale[23]. Ultrasound (US) of the affected knee (Siemens Acuson Antares with a 5–13 MHz linear array transducer) was performed by a single examiner (JC). A standardized protocol based on current guidelines and definitions was followed [24–26]. Knee was scanned in longitudinal and transversal planes with 30° joint flexion. Baseline ultrasound measurements, recorded in millimeters, were: effusion: a ≥4mm hypoechoic or anechoic intraarticular material that was displaceable and compressible in the suprapatellar recess, evaluated using a longitudinal scan; and synovial hypertrophy: a ≥2mm abnormal hypoechoic intraarticular tissue that was non-displaceable and poorly compressible in the suprapatellar recess, measured on a longitudinal scan[27].An intraarticular joint aspiration and glucocorticoid injection were performed as a part of usual clinical practice for these patients with effusion and pain. The US examination, joint aspiration and the IACI (triamcinolone acetonide 40 mg plus lidocaine 1 mg) were performed on the same day of the inclusion visit. The ultrasound findings were dichotomized at one month and at one year follow-up for effusion and synovial hypertrophy (present if ≥4mm and ≥2mm respectively). Synovial fluid was analyzed to ensure non-inflammatory characteristics (white blood cell count ≤1500 cells/mm^3^) and absence of micro-crystals. A blood analysis was carried out during the visit to determine C-reactive protein (CRP) levels. Patients were clinically evaluated at one month and at one year after IACI for the presence of effusion on US and were also asked a question regarding treatment satisfaction. Subjects with enough improvement to carry out normal daily activities with pain VAS <40mm were considered as responders. We performed another IACI at one month or at any time during the one-year follow-up if clinical symptoms reappeared (evaluated as VAS≥40) and effusion was confirmed by US.

### Statistical methods

Clinical data and laboratory parameters were summarized using means and standard deviation (continuous variables), medians and interquartile ranges (continuous asymmetric variables) and frequencies and percentages(categorical variables).Association of numeric variables with group categories were evaluated using a T-test, while a Fisher's or a Chi-square test for contingency tables were used for binary or more than two categories variables, respectively. Due to low sample size in the Kellgren-Lawrence 4 grade group, patients with 3 and 4 grades were combined for analyses; similarly, patients who received four injections were merged with those who received three IACI. Differences in pain and effusion at one year regarding the number of IACI received were assessed using logistic regression.

Logistic regression models were fitted to pain and effusion at one month and at one year, in order to assess independent associations with clinical, anthropometric, radiologic and ultrasound parameters in female patients, controlled and uncontrolled by pain and effusion status at one month. In such models, a backward elimination algorithm for selection of explanatory variables was carried out. In every step, Wald and Hosmer-Lemeshow tests were computed to assess the statistical significance of the coefficients and the goodness of fit of the model, respectively. These models could not be fitted to male patients due to the small number of observations available for analysis. Threshold for statistical significance was set at 5%. All analyses were carried out using StatCrunch.

## Results

One hundred and thirty-two patients were included (characteristics shown in Table 1). Patients mean age was 67.8years and average symptom duration was 55.5months. Mean BMI, WC and percentages of body fat were in the higher clinical range. Only 3 patients (2.3%) were in KOA late stage (KL 4). At one month, thirty-eight patients (28.8%) still reported pain, and the same percentage 38 (28.8%) had persistent effusion. At one year, 51 (38.6%) patients experienced pain and 35 (26.5%) showed effusion.

**Table 1:** Patient characteristics and comparison by gender.

		All patients (n = 132) n (%)/ median SD	Women (n = 111) n (%)/ median SD	Men (n = 21) n (%)/ median SD	*p value*
Age		67.8 (7.9)	68.11 (7.8)	66.6 (8.5)	0.44
OA symptom duration (months)		55.5 (43.4)	57.1 (43.9)	47.4 (40.5)	0.34
BMI (Kg/m2)		31.4 (4.6)	31.3 (4.6)	31.7 (4.7)	0.7
WC (cm)		102.8 (11.5)	101.1 (10.1)	111.9 (14.2)	<0.0001
%Body Fat		40.1 (6.1)	41.8 (4.7)	31.2 (4.5)	<0.0001
Obesity %		73 (55.3%)	62 (55.8%)	11 (53.4%)	0.81
US synovial fluid (mm)#		9.4 (2.7)	9.3 (2.7)	9.9 (2.8)	0.31
US synovial hipertrophy (mm) #		4.3 (2.03)	4.3 (1.9)	4.5 (2.3)	0.64
Blood CRP*		0.4 (0.5)	0.4 (0.4)	0.4 (0.6)	0.43
Synovial fluid cells count*		130 (163)	130 (155)	150 (175)	0.39
Lequesne index		13.5 (3.9)	13.7 (3.9)	12.2 (4.1)	0.13
KL	1	20 (15.2%)	17 (15.3%)	3 (14.3%)	0.75
	2	51 (38.6%)	42 (37.8%)	9 (42.8%)	
	3	58 (43.9%)	49 (44.1%)	9 (42.8%)	
	4	3 (2.3%)	3 (2.7%)	0	
Pain at one month		38 (28.8%)	30 (27.1%)	8 (38.1%)	0.30
US effusion at one month¶		38 (28.8%)	28 (25.2%)	10 (47.6%)	0.03
Pain at one year		51 (38.6%)	41 (36.9%)	10 (47.6%)	0.36
US effusion at one year ¶		35 (26.5%)	28 (25.2%)	7 (33.3%)	0.44.

BMI: Body Mass Index, CRP: C-reactive protein, KL: Kellgren-Lawrence scale, mm: millimeters, OA: Osteoarthritis, SD: standard deviation, US: Ultrasound,WC: Waist Circumference.

Absolute frequencies and percentages were used for categorical variables and means with standard deviation (SD) for continuous variables.

# Baseline measurements expressed in millimeters as a continuous variable.

* Median and interquartile ranges (IQR) were used due to the asymmetry of the distribution.

¶Measurements at one month and one year follow-up, as a dichotomic variable regarding presence or absence.

Table 2 summarizes the associations between different clinical, radiological and ultrasound parameters with pain and effusion at one year. The variables associated to the outcomes and the magnitudes of associations differed across genders. All the univariant associations for pain and effusion at one year and at one month are shown Tables A to D in S1 File.

**Table 2:** Significant associations between the different variables evaluated and pain and effusion at one month and at one year follow-up.

	Variables*		Women N (%)/mean		Men N (%)/mean	
			Yes	No	Yes	No
			*p value*		*p value*	
Pain at one year	Obesity	Yes	29 (70%)	33 (47%)	7 (70%)	4 (36.4%)
		No	12 (30%)	37 (53%)	3 (30%)	7 (63.6%)
			0.0183		0.1984	
	BMI		32.7 (4.38)	30.4 (4.48)	33.3 (5.19)	30.2 (3.91)
			0.009		0.1429	
	WC		104.2 (9.2)	99.3 (10.1)	116.3 (12.8)	107.9 (14.7)
			0.0116		0.1793	
	% Body Fat		43.5 (3.78)	40.7 (4.87)	32.1(4.66)	30.3 (4.33)
			0.002		0.3577	
	Lequesne		15.9 (3.14)	12.3 (3.6)	13.3 (4.11)	11.4 (3.95)
			< 0.0001		0.2853	
	Pain at one month¶	Yes	25 (61%)	5 (7.2%)	7 (70%)	1 (9%)
		No	16 (39%)	65 (92.8%)	3 (30%)	10 (91%)
			< 0.0001		0.0075	
	Effusion at one month¶	Yes	24 (58.5%)	4 (5.7%)	8 (80%)	2 (18.2%)
		No	17 (41.5%)	66 (94.3%)	2 (20%)	9 (81.8%)
			< 0.0001		0.0089	
	Effusion at one yearʺ	Yes	22 (70.9%)	6 (8.5%)	5 (50%)	2 (18.2%)
		No	19 (29.1%)	64 (93.5%)	5 (50%)	9 (81.8%)
			<0.0001		0.1224	
Effusion at one year	WC		104.1 (9.2)	100.1(10.2)	117.2 (11.4)	109.3 (15.1)
			0.0696		0.2413	
	US effusion (mm) #		10.5 (2.85)	8.94 (2.6)	9.81 (2.52)	9.96 (2.96)
			0.0041		0.9101	
	US synovial hypertrophy (mm) #		5.18 (2.2)	3.99 (1.8)	4.04 (1.65)	4.75 (2.59)
			0.0057		0.518	
	Lequesne		15.3 (3.25)	13.1 (3.91)	14.14 (4.37)	11.35 (3.69)
			0.0086		0.1413	
	Pain at one month¶	Yes	15 (53.5%)	15 (18%)	6 (85.7%)	2 (14.2%)
		No	13 (46.5%)	68 (82%)	1 (14.3%)	12 (85.8%)
			0.0005		0.0032	
	Effusion at one month¶	Yes	20 (71.4%)	8 (9.6%)	7 (100%)	3 (21.5%)
		No	8 (28.6%)	75 (90.4%)	0 (0%)	11 (78.5%)
			< 0.0001		0.001	

BMI: Body Mass Index, CI: Confidence Interval, CRP: C-reactive protein, SD: standard deviation, US: Ultrasound, WC: Waist Circumference.

Continuous variables were expressed using means and standard deviation. Comparisons were performed by T-student test (p value reported in the lower line). Categorical variables were expressed by absolute frequencies, percentages and CI of 95%; comparisons were performed using a Fisher’s test (binary variables) or a Chi-square test (for more than two categories).

All variables included in the Table 1 were evaluated in the analyses. In this table, only statistical significant results are shown.

# Baseline measurements expressed in millimeters as a continuous variable.

¶Measurements at one month and one year of follow up, as a dichotomic variable regarding presence or absence.

All the anthropometric measurements, Lequesne index, pain and effusion at one month were associated to pain at one year in women. When effusion was not present at one year, female patients had less probability to experience pain. In men, only the status of pain and effusion at one month was associated to pain at one year. In women the Lequesne index, baseline US effusion, synovial hypertrophy, pain and US effusion at one month were associated to pain at one year, while in men it was only the presence of pain or effusion at one month (Table 2, Tables A and B in S1 File.).

A logistic regression model for effusion and pain at one year in women was performed including the anthropometric, clinical, radiological and ultrasound parameters considered as explanatory (Table 3). The factors independently and directly associated with pain at one year were the Lequesne index (OR 1.36 [1.13–1.64]), % of body fat (OR 1.19 [1.03–1.039]) and the more related variables were pain and effusion at one month (OR 10.46 [2.18–50.06], p 0.0033 and OR 8.37 [1.75–39.93], p 0.0076, respectively). When the status at one month was not considered, the magnitude of association of the Lequesne index and percentage of body fat did not change significantly, pointing to an independent association. For effusion at one year, US synovial hypertrophy and effusion at one month (OR 1.50 [1.09–2.05] p 0.0115 and OR 33.42 [5.84–191.22] p <0.0001, respectively)were the variables directly associated. When the status at one month was not taken into account, the Lequesne index and baseline US synovial hypertrophy were the associated factors pointing to a relationship between the Lequesne index and effusion at one month.

**Table 3:** Adjusted associations with pain and effusion at one year follow-up, evaluating anthropometric measurements, clinical, radiological, ultrasound parameters and the status of pain and effusion at month.

	Variables	Women	
		(OR 95% CI)	*p value*
Pain at one year	% Body Fat	1.19 (1.03–1.39)	0.017
	Blood CRP	0.69 (0.35–1.36)	0.28
	Lequesne	1.36 (1.13–1.64)	0.0012
	Pain at one month	10.46 (2.18–50.06)	0.0033
	Effusion at one month	8.37 (1.75–39.93)	0.0076
Effusion at one year	WC (cm)	1.04 (0.97–1.13)	0.08
	US Synovial hypertrophy	1.50 (1.09–2.05)	0.0115
	Lequesne index	1.09 (0.92–1.29)	0.31
	Effusion at one month	33.42 (5.84–191.22)	<0.0001
	Pain at one month	0.67 (0.12–3.88)	0.66

CI: Confidence Interval, cm: centimetres, CRP: C-reactive protein, US: ultrasound, WC: waist circumference, OR:Odds Ratio.

Due to the significant association between the status of pain and effusion at one year with the status at one month, we evaluated, in women, the factors associated with pain and effusion at one month controlled by anthropometric, clinical, radiological and ultrasound parameters. The Lequesne index was the factor directly and independently associated to the two outcomes and baseline US effusion was associated to effusion at one month. (Table 4).

**Table 4:** Adjusted associations with pain and effusion at one month follow-up, evaluating anthropometric measurements, clinical, radiological and ultrasound parameters.

	Variables	Women	
		(OR 95% CI)	*p value*
Pain at one month	Age	0.95 (0.89–1.01)	0.12
	Blood CRP	1.33 (0.80–2.42)	0.27
	Synovial fluid cell count	0.71 (0.48–1.03)	0.067
	Lequesne index	1.25 (1.09–1.45)	0.0015
Effusion at one month	WC (cm)	0.95(0.89–1.02)	0.189
	%Body Fat	1.14(0.99–1.31)	0.072
	US effusion (mm)	1.18(1.00–1.39)	0.049
	Lequesne index	1.17(1.02–1.35)	0.021

CI: Confidence Interval, cm:centimeters, CRP: C-reactive protein, mm: millimeters, US: ultrasound, WC: waist circumference, OR: Odds Ratio.

The percentage of patients with pain or effusion at one year depending on the number of IACI received and the comparison between the different number of injections and the outcomes are shown in Table 5.

**Table 5:** Comparison of patients experiencing pain and effusion at one year follow-up by number of intraarticular injections received.

	nIACI	Women (n = 111)				Men (n = 21)			
		n (%) IACI received	n (%)*	OR (95% CI)	*p value*	n (%) IACI received	n (%)*	OR (95% CI)	*p value*
Pain at one year	1	59 (53.1%)	12 (20.33%)	Reference		10 (47.6%)	2 (20%)	Reference	
	2	27 (24.3%)	10 (37.03%)	2.30 (0.84–6.30)	0.1039	3 (14.2%)	1 (33.3%)	2 (0.11–34.82)	0.6344
	3	22 (19.8%)	16 (72.7%)	12.40 (4.06–37.8)	<0.0001	5 (23.8%)	4 (80%)	28 (2.06–379.26)	0.0122
	4	3 (2.8%)	3 (100%)			3 (14.4%)	3 (100%)		
Effusion at one year	1	59 (53.1%)	7 (11.8%)	Reference		10 (47.6%)	0 (0%)	Reference	
	2	27 (24.3%)	5 (18.5%)	1.68 (0.48–5.9)	0.412	3 (14.2%)	1 (33.3%)	2.7 (0.16–46.79)	0.484
	3	22 (19.8%)	13 (59.1%)	13.2 (4.24–41.11)	<0.0001	5 (23.8%)	4 (80%)	22 (1.53–314.3)	0.022
	4	3 (2.8%)	3 (100%)			3 (14.4%)	2 (66.7%)		

nIACI: Number of intraarticular corticosteroid injections received, CI: confidence interval, OR: Odds Ratio.

*Patients with pain or effusion at year follow-up depending on the number of IACI received.

Variables expressed by absolute frequencies and percentages for all groups depending on the number of injections received.

The percentage of patients with pain at one year increased depending on the number of IACI received and with the same proportion according to gender (Table 5). In a logistic regression model, we could observe that there were no statistical differences between patients who received one or two IACI, but there were differences in patients who received 3 or 4 compared to one IACI (women: p<0.0001/ men: p 0.0122). The percentage of patients with US effusion also increased depending on the number of IACI performed (Table 5). No statistical differences between patients who received one or two IACI were observed in the logistic regression model, buteffusion was detected more frequently in patients receiving 3–4 IACI compared to one when analyzed separately by gender (women: p<0.0001/men: p 0.022), which could be considered as non responders.

## Discussion

In this pragmatic study set in clinical practice, our objective was to analyze the effect of IACI in patients with knee OA with persistent joint effusion following a specific preestablished protocol of treatment based on repeating the IACI when significant pain and effusion by US were present. Our results indicate that IACI could be a good treatment option for these patients as 61.4% remained with no pain at one year[28], and the effect appeared to be better in women than in men (63.1% vs. 52.4%), although it is difficult to draw conclusions about these differences due to the small sample of men.

Based on our results, one IACI could be strongly recommended for this type of patients. For patients responding at one month, there was a high probability of response in case of an additional IACI due to an inflammatory flare. However, patients with persisting pain or effusion at one month after IACI showed a minimal probability to respond to additional injection. So, according to our observations it could be inferred that the effect evaluated at one month could predict the response at one year with the protocol applied in this study.

Little data exist in the literature about response to IACI and the studies have poor follow-up times[29–33]. Some previous studies have suggested a better response in patients with a milder radiographic OA[31] or with joint effusion[17, 31], but all our patients had effusion and we did not observe differences between radiographic degrees. Previous studies concluded benefit of IACI at four weeks, a lack of evidence from 4 to 24 weeks and no benefit after 24 weeks of IACI administration[10, 33]. However, these studies did not take into account the presence of effusion. In fact, to our knowledge, only one study analyzed the IACI effect in knee OA at one year. This randomized controlled trial evaluating the safety and efficacy of a IACI repeated every three months observed no differences between placebo and IACI groups regarding pain or radiologic progression at one- and two-year follow-up[34]. In our study patients receiving more IACI did not have a better response at one year if did not improve at one month. On the other hand, patients responding at one month benefit from additional IACI if needed. So, in our opinion, our results and the review of previous studies suggest a benefit of IACI in knee OA with effusion and the effect at one month could point to a better responder subgroup.

In women, the Lequesne index appeared to be the most related parameter independently associated to pain at one month. Synovial fluid cell count appeared to have an inverse effect, with an inferior proportion of patients with fewer synovial fluid cells experiencing a good response to IACI, although it did not reach statistical significance. Regarding pain at one year, the Lequesne index and the percentage of body fat were independently associated, so that patients with higher scores in the Lequesne index and higher body fat percentage showed less probability to respond to IACI. Therefore, in our group of patients with knee OA and joint effusion, the Lequesne index and body fat percentage could serve as factors predicting response to IACI at one month and at one year.

In women, the Lequesne index and the amount of US synovial fluid at the time of IACI were independently associated to persistent effusion at one month. At one year, the parameters independently associated to US effusion were the Lequesne index and US synovial hypertrophy at baseline. Thus, in our group of patients with inflammatory features, having more pain and more US parameters of inflammation were associated to a greater presence of effusion at one year of follow-up.

Pain and effusion at one month were closely and independently related to pain at one year, while effusion at one month was associated with effusion at one year. These parameters could serve as predictors of response during follow-up and highlight the importance of monitoring response after one month to determine future IACI management.

In our group, pain was closely related to the presence of US effusion, so that we could deduce that the parameters associated to persistence of effusion were partially related to pain at one year. Different previous studies have tried to identify some predictor of good response to IACI [35, 36]. Some of the data suggest that patients with effusion or inflammatory parameters could obtain more benefit from IACI[17]. In our group, all patients had effusion at the moment of IACI and they were non-responders to other therapies. Therefore, we could not evaluate if effusion was a parameter of good response. Other authors have tried to identify signs of inflammation on ultrasound as predictors for response, but after four weeks no reliable and clinically meaningful predictor was found [27, 37, 38]. We did not find ultrasound parameters directly associated to pain, but ultrasound measures in our study were associated to effusion during follow-up. In accordance to previous data, little association was found at one month in our study[27], but synovial hypertrophy was closely associated to persistent effusion at one year. Some previous data found no association between synovitis or joint effusion as predictors of response to IACI in knee OA patients, with longer benefit from IACI in patients with less severe signs of inflammation [37, 38], consistent with our observation that patients with more synovial hypertrophy had more ultrasound-detected effusion at one year. A recent meta-analysis found and association between major improvement in short-term pain and more severe pain at baseline but without differences regarding mid- and long-term pain[39]. We found that patients with more severe clinical disease improved less. However, our definition of improvement differs from previous studies, and was not based on a mean change between questionnaire results, that sometimes are not clinically significant, but it was based on the patient’s opinion about their improvement, which may better reflect a clinically significant improvement. In contrast, in our study we observed differences in long-term pain. Another important aspect is that patients with greater clinical severity, measured as high scores in the Lequesne index, could be more affected by pain sensitization[40], as this appears to be associated with joint effusion, as patients in our group[41]. Thus, patients with high scores in the Lequesne index could not be good candidates for a local pain technique such as IACI, unless this point warrants more study. Different data exists in the literature supporting the negative effect of body fat mass or obesity in knee OA[42, 43], but to our knowledge, this is the first time when percentage of body fat mass has been found to be associated to pain at one year. Differences in gender exist in the literature regarding pain in knee OA [44, 45]. The profile of inflammatory molecules like adipokines that might participate in knee effusion and OA-related inflammation are different across genders[46]. Thus, the response profile to IACI could also be different in relation to gender, and the results of our study clearly suggest this possibility, although the low number of men in this sample precludes definite conclusions.

Our study has some strengths and limitations. The main strength is the homogeneity of the sample, which increases the statistical power to detect associations. All patients were recruited from general clinical practice using the referrals to our Rheumatology Service and not collected from Hospital databases that could have selected a more severe disease. The main limitation arises from its observational nature and therefore its inability to establish causality, so that only associations could be drawn. This was not a randomized clinical trial, all patients were treated and no controlled placebo arm was available. Patients answered a validated questionnaire at the moment of IACI but not during follow-up, and response criteria were established clinically as defined in methods. We understand that the lack of a validated questionnaire to evaluate response is a limitation of our study but, in our opinion, a dichotomic question regarding response is a hard endpoint because it is a more straightforward question. Ultrasound evaluations were made only by one experienced rheumatologist as it was a study carried out in daily clinical practice and we have no intra-observer agreement index. On the other hand, US measures were consistent, as all US exams were performed by the same explorer. Lastly, the lack of adequate collection of symptomatic drugs for OA is also a limitation. However, all patients in our group were symptomatic and were taking pain-relief medication.

In conclusion, in this pragmatic study we have tried to identify clinical parameters at the moment of first IACI associated to clinical response in knee OA patients with joint effusion. The Lequesne index and body fat percentage were associated to pain at one year, and synovial hypertrophy was related to the presence of effusion at one year. These patients benefited from IACI, and the response at one month could determine a more effective response at one year and to select candidates for additional IACI. Our observations warrant replication and evaluation in a randomized clinical trial to investigate the effect of IACI.

## Supporting information

S1 File **Table A. Associations between the different variables evaluated and pain at one monthof follow up.** BMI: Body Mass Index, CI: Confidence Interval, CRP: C-reactive protein, SD: standard deviation, US: Ultrasound, WC: Waist Circumference. Continuous variables were expressed using means and standard deviation. Comparisons were performed by T-student test (p value reported in the lower line). Categorical variables were expressed by absolute frequencies, percentages and CI of 95%; comparisons were performed using a Fisher’s test (binary variables) or a Chi-square test (for more than two categories). # Baseline measurements expressed in millimeters as a continuous variable.^Variables were log transformed to realize the analysis, ¶Measurements at one month and one year of follow up, as a dichotomic variable regarding presence or absence.**Table B. Associations between the different variables evaluated and effusion at one monthof follow up.** BMI: Body Mass Index, CI: Confidence Interval, CRP: C-reactive protein, SD: standard deviation, US: Ultrasound, WC: Waist Circumference. Continuous variables were expressed using means and standard deviation. Comparisons were performed by T-student test (p value reported in the lower line). Categorical variables were expressed by absolute frequencies, percentages and CI of 95%; comparisons were performed using a Fisher’s test (binary variables) or a Chi-square test (for more than two categories). # Baseline measurements expressed in millimeters as a continuous variable.^Variables were log transformed to realize the analysis.**Table C. Associations between the different variables evaluated and pain at one year of follow up.** BMI: Body Mass Index, CI: Confidence Interval, CRP: C-reactive protein, SD: standard deviation, US: Ultrasound, WC: Waist Circumference. Continuous variables were expressed using means and standard deviation. Comparisons were performed by T-student test (p value reported in the lower line). Categorical variables were expressed by absolute frequencies, percentages and CI of 95%; comparisons were performed using a Fisher’s test (binary variables) or a Chi-square test (for more than two categories). # Baseline measurements expressed in millimeters as a continuous variable.^Variables were log transformed to realize the analysis, ¶Measurements at one month and one year of follow up, as a dichotomic variable regarding presence or absence.**Table D. Associations between the different variables evaluated and effusion at one year of follow up.** BMI: Body Mass Index, CI: Confidence Interval, CRP: C-reactive protein, SD: standard deviation, US: Ultrasound, WC: Waist Circumference. Continuous variables were expressed using means and standard deviation. Comparisons were performed by T-student test (p value reported in the lower line). Categorical variables were expressed by absolute frequencies, percentages and CI of 95%; comparisons were performed using a Fisher’s test (binary variables) or a Chi-square test (for more than two categories). # Baseline measurements expressed in millimeters as a continuous variable.^Variables were log transformed to realize the analysis, ¶Measurements at one month and one year of follow up, as a dichotomic variable regarding presence or absence.
(DOCX)
