# Supplementary material for: Clinical and ultrasonographic features associated to response to intraarticular corticosteroid injection. A one year follow up prospective cohort study in knee osteoarthritis patient with joint effusion
Source: PLoS One. 2018 Jan 19;13(1):e0191342. doi: 10.1371/journal.pone.0191342 (PMC5774783; doi:10.1371/journal.pone.0191342)
Supplement: S1 File — Table A. Associations between the different variables evaluated and pain at one monthof follow up. BMI: Body Mass Index, CI: Confidence Interval, CRP: C-reactive protein, SD: standard deviation, US: Ultrasound, WC: Waist Circumference. Continuous variables were expressed using means and standard deviation. Comparisons were performed by T-student test (p value reported in the lower line). Categorical variables were expressed by absolute frequencies, percentages and CI of 95%; comparisons were performed using a Fisher’s test (binary variables) or a Chi-square test (for more than two categories). # Baseline measurements expressed in millimeters as a continuous variable.^Variables were log transformed to realize the analysis, ¶Measurements at one month and one year of follow up, as a dichotomic variable regarding presence or absence.Table B. Associations between the different variables evaluated and effusion at one monthof follow up. BMI: Body Mass Index, CI: Confidence Interval, CRP: C-reactive protein, SD: standard deviation, US: Ultrasound, WC: Waist Circumference. Continuous variables were expressed using means and standard deviation. Comparisons were performed by T-student test (p value reported in the lower line). Categorical variables were expressed by absolute frequencies, percentages and CI of 95%; comparisons were performed using a Fisher’s test (binary variables) or a Chi-square test (for more than two categories). # Baseline measurements expressed in millimeters as a continuous variable.^Variables were log transformed to realize the analysis.Table C. Associations between the different variables evaluated and pain at one year of follow up. BMI: Body Mass Index, CI: Confidence Interval, CRP: C-reactive protein, SD: standard deviation, US: Ultrasound, WC: Waist Circumference. Continuous variables were expressed using means and standard deviation. Comparisons were performed by T-student test (p value reported in the lower line). Categorical variables were expres [file pone.0191342.s001.docx]

Table A. Associations between the different variables evaluated and pain at one month of follow up.

| Variables* | | Women  N (% and CI 95%)/mean | | Men  N (% and CI 95%)/mean | |
| --- | --- | --- | --- | --- | --- |
|  | | Yes | No | Yes | No |
|  |  | *p value* | | *p value* | |
| Age | | 67 (7.5) | 68.5 (7.9) | 66.2 (7.5) | 66.9 (9.2) |
|  |  | 0.36 | | 0.86 | |
| OA symptoms duration (months | | 49.6 (38.4) | 59.8 (45.7) | 49 (32.7) | 46.3 (45.9) |
|  |  | 0.67 | | 0.89 | |
| BMI (kg/m2) | | 31.9 (4.5) | 31.1 (4.6) | 33.8 (4.3) | 30.3 (4.7) |
|  |  | 0.395 | | 0.107 | |
| WC (cm) | | 102.95 (9.85) | 100.43 (10.1) | 119.7 (11.3) | 107.2 (13.69) |
|  |  | 0.24 | | 0.045 | |
| % Body Fat | | 42.4 (3.9) | 41.5 (4.9) | 33.4 (2.8) | 29.8 (4.8) |
|  |  | 0.34 | | 0.07 | |
| Obesity % | | 18 (29%) (17.7-40.3) | 44 (71%)  (59.7-82.3) | 7 (63.6%)  (35.2-92.1) | 4 (36.4%) (7.9-64.8) |
|  |  | 0.6696 | | 0.0237 | |
| US synovial fluid # | | 9.5 (3.0) | 9.1 (2.6) | 10.8 (2.3) | 9.3 (2.9) |
|  |  | 0.489 | | 0.213 | |
| US synovial hipertrophy # | | 4.1 (1.7) | 4.3 (2.1) | 4.9 (2.2) | 4.3 (2.4) |
|  |  | 0.6 | | 0.6 | |
| Log Blood CRP ^ | | 1.16 (0.96) | 0.98 (0.75) | 1.26 (0.93) | 0.50 (0.94) |
|  |  | 0.375 | | 0.084 | |
| Synovial fluid cells count^ | | 4.5 (1.6) | 4.7 (1.1) | 4.9 (1.1) | 5.1 (1.1) |
|  |  | 0.44 | | 0.91 | |
| Lequesne index | | 15.43 (3.5) | 13.02 (3.8) | 14.87 (3.3) | 10.69 (3.7) |
|  |  | 0.0032 | | 0.017 | |
| KL | 1 | 13 (76.5%)  (53.6-96.6) | 4 (23.5%)  (3.4-43.7) | 3 (100%) | 0 (0%) |
|  | 2 | 33 (78.5%)  (66.2-91) | 9 (21.5%)  (9-33.8) | 5 (55.5%)  (23.1-88) | 4 (44.5%)  (12-76.9) |
|  | 3-4 | 35 (67.3%)  (54.6-80.1) | 17 (32.7%)  (19.9-45.4) | 5 (55.5%)  (23.1-88) | 4 (44.5%)  (12-76.9) |
|  |  | 0.45 | | 0.34 | |
| US Effusion at one month ¶ | | 21 (75%)  (59.0-91.0) | 7 (25%)  (9.0-41.0) | 8 (80%)  (55.2-100) | 2 (20%)  (0-44.8) |
|  |  | < 0.0001 | | 0.0002 | |

BMI: Body Mass Index, CI: Confidence Interval, CRP: C-reactive protein, SD: standard deviation, US: Ultrasound, WC: Waist Circumference.

Continuous variables were expressed using means and standard deviation. Comparisons were performed by T-student test (p value reported in the lower line). Categorical variables were expressed by absolute frequencies, percentages and CI of 95%; comparisons were performed using a Fisher’s test (binary variables) or a Chi-square test (for more than two categories).

# Baseline measurements expressed in millimeters as a continuous variable. ^Variables were log transformed to realize the analysis, ¶Measurements at one month and one year of follow up, as a dichotomic variable regarding presence or absence.

Table B. Associations between the different variables evaluated and effusion at one month of follow up.

| Variables* | | Women  N (% and CI 95%)/mean | | Men  N (% and CI 95%)/mean | |
| --- | --- | --- | --- | --- | --- |
|  | | Yes | No | Yes | No |
|  |  | *p value* | | *p value* | |
| Age | | 68.2 (7.1) | 68 (8.1) | 65.8 (7.9) | 67.4 (9.2) |
|  |  | 0.89 | | 0.66 | |
| OA symptoms duration (months | | 57.6 (51.4) | 56.9 (41.5) | 44.2 (30.3) | 50.2 (49.2) |
|  |  | 0.91 | | 0.74 | |
| BMI (kg/m2) | | 31.7 (4.3) | 31.2 (4.7) | 33.8 (4.7) | 29.6 (3.9) |
|  |  | 0.607 | | 0.045 | |
| WC (cm) | | 102.76 (8.41) | 100.55 (10.5) | 119.5 (10.68) | 105 (13.72) |
|  |  | 0.3155 | | 0.0144 | |
| % Body Fat | | 43 (3.3) | 41.3 (5.1) | 32.9 (3.2) | 29.5 (4.9) |
|  |  | 0.1 | | 0.08 | |
| Obesity % | | 15 (24.2%)  (13.5-34.9) | 47 (75.8%)  (65.1-86.5) | 8 (72.7%)  (46.4-99.0) | 3 (27.3%)  (1.0-53.6) |
|  |  | 0.7783 | | 0.03 | |
| US synovial fluid # | | 10.15 (2.82) | 8.94 (2.64) | 10.62 (2.51) | 9.27 (2.93) |
|  |  | 0.0426 | | 0.275 | |
| US synovial hipertrophy # | | 4.6 (2.1) | 4.1 (1.9) | 4.8 (2.2) | 4.2 (2.4) |
|  |  | 0.25 | | 0.51 | |
| Blood CRP ^ | | 1.2 (0.8) | 1.1 (0.9) | 0.7 (1) | 1.2 (0.9) |
|  |  | 0.68 | | 0.28 | |
| Synovial fluid cells count^ | | 4.8 (1.3) | 4.6 (1.2) | 4.9 (0.93) | 5.1 (1.2) |
|  |  | 0.41 | | 0.74 | |
| Lequesne | | 15.21 (3.61) | 13.15 (3.83) | 13.5 (4.08) | 11.18 (3.86) |
|  |  | 0.0143 | | 0.197 | |
| KL | 1 | 16 (94.1%)  (82.9-100) | 1 (5.9%)  (0-17.1) | 2 (66.6%)  (13-100) | 1 (33.4%)  (0-86.6) |
|  | 2 | 30 (71.4%)  (57.8-85.1) | 12 (28.6%)  14.9-42.2) | 4 (44.5%)  (12-76.9) | 5 (55.5%)  (23.1-88) |
|  | 3-4 | 37 (71.1%)  (58.8-83.5) | 15 (28.9%)  (16.5-41.2) | 5 (55.5%)  (23.1-88) | 4 (44.5%)  (12-76.9) |
|  |  | 0.14 | | 0.77 | |

BMI: Body Mass Index, CI: Confidence Interval, CRP: C-reactive protein, SD: standard deviation, US: Ultrasound, WC: Waist Circumference.

Continuous variables were expressed using means and standard deviation. Comparisons were performed by T-student test (p value reported in the lower line). Categorical variables were expressed by absolute frequencies, percentages and CI of 95%; comparisons were performed using a Fisher’s test (binary variables) or a Chi-square test (for more than two categories).

# Baseline measurements expressed in millimeters as a continuous variable. ^Variables were log transformed to realize the analysis.

Table C. Associations between the different variables evaluated and pain at one year of follow up.

| Variables* | | Women  N (% and CI 95%)/mean | | | Men  N (% and CI 95%)/mean | |
| --- | --- | --- | --- | --- | --- | --- |
|  | | Yes | | No | Yes | No |
|  |  | *p value* | | | *p value* | |
| Age | | 68.9 (7.2) | | 67.6 (7.2) | 67.4 (8) | 66 (9.2) |
|  |  | 0.407 | | | 0.71 | |
| OA symptoms duration (months | | 61.1 (46.9) | | 54.6 (42.2) | 60.8 (43.2) | 35.1 (35.4) |
|  |  | 0.45 | | | 0.15 | |
| BMI (kg/m2) | | 32.7 (4.38) | | 30.4 (4.48) | 33.3 (5.19) | 30.2 (3.91) |
|  |  | 0.009 | | | 0.1429 | |
| WC (cm) | | 104.2 (9.2) | | 99.3 (10.1) | 116.3 (12.8) | 107.9 (14.7) |
|  |  | 0.0116 | | | 0.1793 | |
| % Body Fat | | 43.5 (3.78) | | 40.7 (4.87) | 32.1(4.66) | 30.3 (4.33) |
|  |  | 0.002 | | | 0.3577 | |
| Obesity % | | 29 (46.8%)  (34.4-59.2) | | 33 (53.2%)  (40.8-65.6) | 7 (63.6%)  (35.2-92.1) | 4 (36.4%)  (7.9-64.8) |
|  |  | 0.0183 | | | 0.1984 | |
| US synovial fluid # | | 9.7 (2.8) | | 8.9 (2.6) | 10.2 (3.1) | 9.6 (2.5) |
|  |  | 0.16 | | | 0.67 | |
| US synovial hipertrophy # | | 4.4 (2.1) | | 4.2 (1.9) | 4.5 (2.5) | 4.5 (2.2) |
|  |  | 0.68 | | | 0.94 | |
| Blood CRP ^ | | 1.1 (0.92) | | 1.1 (0.91) | 0.97 (1.05) | 0.96 (0.97) |
|  |  | 0.91 | | | 0.98 | |
| Synovial fluid cells count^ | | 4.8 (1.6) | | 4.6 (0.96) | 5.03 (1.2) | 5.01 (0.9) |
|  |  | 0.417 | | | 0.97 | |
| Lequesne index | | 15.9 (3.14) | | 12.3 (3.6) | 13.3 (4.11) | 11.4 (3.95) |
|  |  | < 0.0001 | | | 0.2853 | |
| KL | 1 | 13 (76.5%)  (53.6-96.6) | | 4 (23.5%)  (3.4-43.7) | 2 (66.6%)  (13-100) | 1 (33.3%)  (0-86.6) |
|  | 2 | 26 (62%)  (47.2-76.6) | | 16 (32%)  23.4-52.8) | 6 (66.6 %)  (35.9-97.5) | 3 (33.3%)  (2.5-64.1) |
|  | 3-4 | 31 (59.6%)  (46.3-73) | | 21 (40.4%)  (27-53.7) | 3 (33.3%)  (2.5-64.1) | 6 (66.6%)  (35.9-97.5) |
|  |  | 0.45 | | | 0.32 | |
| Effusion at one month¶ | | 24 (85.7%)  (72.8-98.7) | | 4 (14.3%)  (1.3-27.2) | 8 (80%)  (55.2-100) | 2 (20%)  (0-44.8) |
|  |  | < 0.0001 | | | 0.0089 | |
| Pain at one month¶ | | 25 (83.3%)  (70.0-96.7) | 5 (16.7%)  (3.3-30.0) | | 7 (87.5%)  (64.6-100) | 1 (12.5%)  (0-35.4) |
|  |  | < 0.0001 | | | 0.0075 | |
| Effusion at one year¶ | | 22 (78.6%)  (63.4-93.8) | 6 (21.4%)  (6.2-36.6) | | 5 (71.4%)  (38.0-100) | 6 (28.6%)  (0-62.0) |
|  |  | <0.0001 | | | 0.1224 | |

BMI: Body Mass Index, CI: Confidence Interval, CRP: C-reactive protein, SD: standard deviation, US: Ultrasound, WC: Waist Circumference.

Continuous variables were expressed using means and standard deviation. Comparisons were performed by T-student test (p value reported in the lower line). Categorical variables were expressed by absolute frequencies, percentages and CI of 95%; comparisons were performed using a Fisher’s test (binary variables) or a Chi-square test (for more than two categories).

# Baseline measurements expressed in millimeters as a continuous variable. ^Variables were log transformed to realize the analysis, ¶Measurements at one month and one year of follow up, as a dichotomic variable regarding presence or absence.

Table D. Associations between the different variables evaluated and effusion at one year of follow up.

| Variables* | | Women  N (% and CI 95%)/mean | | | Men  N (% and CI 95%)/mean | |
| --- | --- | --- | --- | --- | --- | --- |
|  | | Yes | | No | Yes | No |
|  |  | *p value* | | | *p value* | |
| Age | | 68.8 (7.6) | | 67.9 (7.9) | 68.6 (6.7) | 65.5 (9.2) |
|  |  | 0.61 | | | 0.42 | |
| OA symptoms duration (months | | 64.2 (50.6) | | 54.6 (41.4) | 47.4 (36.2) | 47.3 (43.8) |
|  |  | 0.31 | | | 0.99 | |
| BMI (kg/m2) | | 32.5 (5.1) | | 30.9 (4.1) | 31.6 (3.5) | 31.8 (5.4) |
|  |  | 0.11 | | | 0.93 | |
| WC (cm) | | 104.1 (9.2) | | 100.1 (10.2) | 117.2 (11.4) | 109.3 (15.1) |
|  |  | 0.0696 | | | 0.2413 | |
| % Body Fat | | 42.7 (3.8) | | 41.4 (4.9) | 32.1 (3.5) | 30.7 (4.9) |
|  |  | 0.22 | | | 0.5 | |
| Obesity % | | 46 (74.2%)  (63.3-85.1) | | 16 (25.8%)  (14.9-36.7) | 6 (54.5%)  (25.1-84) | 5 (45.5%)  (16-74.9) |
|  |  | 0.87 | | | 0.21 | |
| US synovial fluid # | | 10.5 (2.85) | | 8.94 (2.6) | 9.81 (2.52) | 9.96 (2.96) |
|  |  | 0.0041 | | | 0.9101 | |
| US synovial hipertrophy # | | 5.18 (2.2) | | 3.99 (1.8) | 4.04 (1.65) | 4.75 (2.59) |
|  |  | 0.0057 | | | 0.518 | |
| Blood CRP ^ | | 1.15 (0.95) | | 1.1 (0.9) | 0.67 (1.2) | 1.12 (0.87) |
|  |  | 0.802 | | | 0.34 | |
| Synovial fluid cells count^ | | 4.8 (1.3) | | 4.6 (1.2) | 5.03 (1) | 5.02 (1) |
|  |  | 0.41 | | | 0.97 | |
| Lequesne index | | 15.3 (3.25) | | 13.1 (3.91) | 14.14 (4.37) | 11.35 (3.69) |
|  |  | 0.0086 | | | 0.1413 | |
| KL | 1 | 17 (100%) | | 0 (0%) | 3 (100%) | 0 (0%) |
|  | 2 | 31 (73.8%)  (60.5-87.1) | | 11 (26.2%)  12.9-39.5) | 4 (44.5%)  (12-76.9) | 5 (55.5%)  (23.1-88) |
|  | 3-4 | 35 (67.3%)  (54.6-80.1) | | 17 (32.7%)  (19.9-45.4) | 7 (77.7%)  (50.6-100) | 2 (22.3%)  (0-49.5) |
|  |  | 0.02 | | | 0.14 | |
| Effusion at one month¶ | | 20 (71.4%)  (54.7-88.2) | | 8 (28.6%)  (11.8-45.3) | 7 (70%)  (41.6-98.4) | 3 (30%)  (1.6-58) |
|  |  | < 0.0001 | | | 0.001 | |
| Pain at one month¶ | | 15 (53.6%)  (35.1-72.0) | 13 (46.4%)  (28.0-64.9) | | 6 (85.7%)  (59.8-100) | 1 (14.3%)  (31.0-73.7) |
|  |  | 0.0005 | | | 0.0032 | |

BMI: Body Mass Index, CI: Confidence Interval, CRP: C-reactive protein, SD: standard deviation, US: Ultrasound, WC: Waist Circumference.

Continuous variables were expressed using means and standard deviation. Comparisons were performed by T-student test (p value reported in the lower line). Categorical variables were expressed by absolute frequencies, percentages and CI of 95%; comparisons were performed using a Fisher’s test (binary variables) or a Chi-square test (for more than two categories).

# Baseline measurements expressed in millimeters as a continuous variable. ^Variables were log transformed to realize the analysis, ¶Measurements at one month and one year of follow up, as a dichotomic variable regarding presence or absence.
